# Planar hyperbolic polaritons in 2D van der Waals materials

**DOI:** 10.1038/s41467-023-43992-8

**Published:** 2024-01-02

**Authors:** Hongwei Wang, Anshuman Kumar, Siyuan Dai, Xiao Lin, Zubin Jacob, Sang-Hyun Oh, Vinod Menon, Evgenii Narimanov, Young Duck Kim, Jian-Ping Wang, Phaedon Avouris, Luis Martin Moreno, Joshua Caldwell, Tony Low

**Affiliations:** 1 Department of Electrical and Computer Engineering, University of Minnesota, Minneapolis, MN 55455 USA; 2 Institute of High Pressure Physics, School of Physical Science and Technology, Ningbo University, 315211 Ningbo, China; 3 Laboratory of Optics of Quantum Materials, Department of Physics, IIT Bombay, Mumbai, Maharashtra 400076 India; 4 Department of Mechanical Engineering, Materials Research and Education Center, Auburn University, Auburn, AL 36849 USA; 5 Interdisciplinary Center for Quantum Information, State Key Laboratory of Extreme Photonics and Instrumentation, ZJU-Hangzhou Global Science and Technology Innovation Center, College of Information Science and Electronic Engineering, Zhejiang University, 310027 Hangzhou, China; 6 Birck Nanotechnology Center, School of Electrical and Computer Engineering, Purdue University, West Lafayette, IN 47907 USA; 7 Department of Physics, City College and Graduate Center, City University of New York, New York, NY 10031 USA; 8 Department of Physics and Department of Information Display, Kyung Hee University, Seoul, 02447 Republic of Korea; 9 IBM T. J. Watson Research Center, Yorktown Heights, NY 10598 USA; 10 Instituto de Nanociencia y Materiales de Aragon (INMA), CSIC-Universidad de Zaragoza, Zaragoza, 50009 Spain; 11 Departamento de Fisica de la Materia Condensada, Universidad de Zaragoza, Zaragoza, 50009 Spain; 12 Department of Mechanical Engineering, Vanderbilt University, Nashville, TN 37235 USA

**Keywords:** Nanophotonics and plasmonics, Metamaterials, Polaritons

## Abstract

Anisotropic planar polaritons - hybrid electromagnetic modes mediated by phonons, plasmons, or excitons - in biaxial two-dimensional (2D) van der Waals crystals have attracted significant attention due to their fundamental physics and potential nanophotonic applications. In this Perspective, we review the properties of planar hyperbolic polaritons and the variety of methods that can be used to experimentally tune them. We argue that such natural, planar hyperbolic media should be fairly common in biaxial and uniaxial 2D and 1D van der Waals crystals, and identify the untapped opportunities they could enable for functional (i.e. ferromagnetic, ferroelectric, and piezoelectric) polaritons. Lastly, we provide our perspectives on the technological applications of such planar hyperbolic polaritons.

## Abstract (web summary)

In this Perspective, the authors illustrate the physics of hyperbolic polaritons in anisotropic 2D and 1D materials, proposing new potential material candidates, forward looking opportunities and technological applications.

## Introduction

**Fig. 1:**
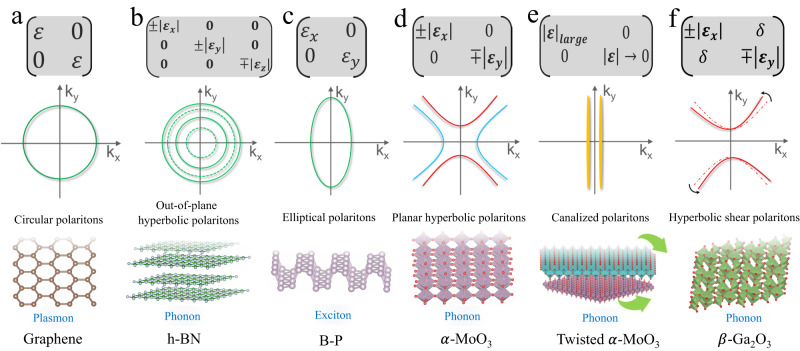
Archetypal van der Waals (vdW) materials hosting polaritonic excitations, their propagation characteristics, and dielectric tensors. Schematic shows isofrequency contours of circular polaritons in **a** graphene, **b** out-of-plane hyperbolic polaritons in h-BN, **c** elliptical polaritons in B-P, **d** planar hyperbolic polaritons in *α*-MoO_3_, **e** canalized polaritons in twisted *α*-MoO_3_, and **f** shear hyperbolic polaritons in *β*-Ga_2_O_3_. The dielectric tensors required to induce the aforementioned polaritons are presented for each of the 2D materials. *ϵ*_*x*_ and *ϵ*_*y*_ represent in-plane dielectric components along the **x** and **y** directions, and *ϵ*_*z*_ is out-of-plane dielectric component along the **z** direction. The alternating solid and dashed isofrequency contour lines in panel **b** serve to highlight the propagation of out-of-plane hyperbolic polaritons.

Polaritons are a hybrid of electromagnetic (EM) modes and dipolar excitations, possessing dispersion characteristics that are intermediate between those of the collective excitations of the material and photons^1^. These polaritons are essential in the fundamental physics and practical applications of light–matter interactions^2^. Examples of these modes include plasmon, exciton, and phonon polaritons, which have become critical elements of both the standard theoretical description as well as practical applications in photonics and optoelectronics^3,4^. Of particular importance, there are the surface polaritonic modes. In general, most existing materials support two-dimensional polaritons that have a two-dimensional (2D) isotropic isofrequency contour, thus energy flows in all directions in the plane. However, many applications would greatly benefit from EM modes that possess highly directional and broadband subdiffractional confinement. The resulting search for novel polaritonic modes in new materials lead to the discovery and development of a new class of anisotropic 2D materials that naturally possess such EM modes, in the form of plasmon, exciton and phonon polaritons. Figure 1 illustrates the change of the polaritonic character from the isotropic circular plasmon polariton in graphene^5^, to the out-of-plane hyperbolic phonon polariton in h-BN^6^, the elliptical plasmon and exciton polariton in black phosphorus^7,8^, and even with a change in topology to the planar hyperbolic phonon polariton in *α*-MoO_3_^9^, and its canalized and shear variant in its twisted structures and beyond^10,11^. Particularly important in this class are the hyperbolic polaritons, due to their inherent singularity in the density of states^12^ and resulting enhancement of light–matter interactions.

Here we discuss the origin of these planar hyperbolic polariton modes, their experimental observations as well as the variety of ways by which their polaritonic properties can be tuned through fabricating heterostructures, applying strain, or utilizing electrostatic gates, intercalation, nanostructuring, hypercrystals and image polaritons. We also review the plethora of novel properties of planar hyperbolic polaritons, such as the unique ray-like propagation, and magic angles corresponding to topological transitions, shear polaritons, longitudinal plasmon spins, and chiral plasmons. Lastly, we provide our perspectives on possible opportunities that new hyperbolic materials with properties, such as ferromagnetic, ferroelectric, and piezoelectric, may offer to fields such as quantum and spin photonics, thermal management, sensing, subwavelength focusing, transformation optics, and polarization engineering.

## Hyperbolic polaritons in flatland

### Directionally confined EM waves

We shall begin with a brief historical account of directionally confined EM waves. Due to symmetry, directional EM waves can only exist in anisotropic media. The interest in anisotropic dielectrics as a means to confine and collimate light was boosted after Dyakonov found in 1988^13^ that the interface between a non-magnetic isotropic dielectric and a non-magnetic uniaxial crystal supports, under specific conditions, a bound EM mode, known now as a Dyakonov wave (DW). The existence of DW requires stringent conditions on the orientation of the optic axis with respect to the interface, propagation angle of the wave and of the relative values of all of the dielectric constants involved. This combination of demanding dielectric requirements and reduced cone directions made the experimental confirmation of the existence of DWs^14^ elusive for some time.

An interesting variation occurs when the dielectric semi-infinite region is replaced by a metal, with the other semi-infinite medium still being a non-magnetic uniaxial crystal. Bound EM fields exist in this case and are denoted as Dyakonov plasmons^15^. Dyakonov plasmons occur within a finite frequency range and only over a narrow range of propagation angles, leading to an open isofrequency curve shaped like a hyperbola. Using these properties, the hyperbolic-like isofrequency curve was used to demonstrate hyperlensing in a system where the uniaxial crystal was a metamaterial^16^.

**Fig. 2:**
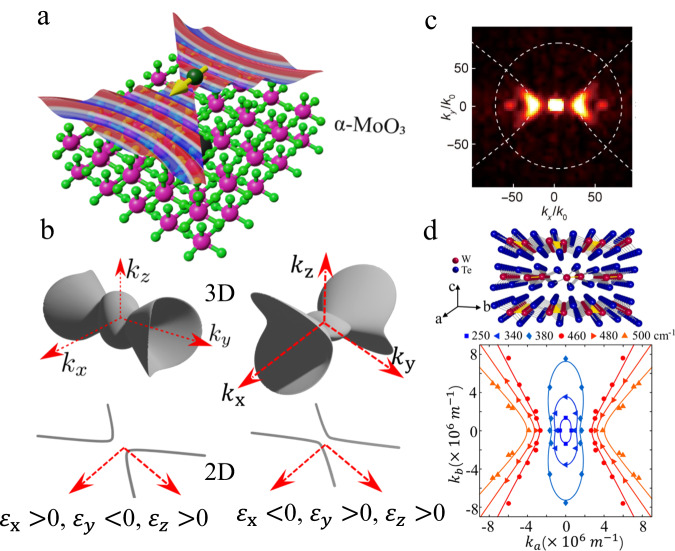
First experimental demonstration of natural planar hyperbolic phonon and plasmon polaritons. **a** The first 2D hyperbolic polariton demonstration was the phonon polariton in *α*-MoO_3_, for which the wavefronts are shown here. **b** 3D and 2D isofrequency surfaces of hyperbolic phonon polaritons in vdW *α*-MoO_3_. *k*_*x*_, *k*_*y*_, and *k*_*z*_ represent wave vectors along the three principal axes. **c** Absolute values of the Fourier transform near-field images of hyperbolic phonon polaritons recorded at 944 cm^−1^ in vdW *α*-MoO_3_ flake, where *k*_0_ denotes the wave vector of free-space. **d** A demonstration of plasmonic 2D hyperbolic polariton. Isofrequency contours of plasmon at different frequencies are indicative of the topological transition in WTe_2_ thin films. *k*_*a*_ and *k*_*b*_ denote wave vectors along **a** and **b** axes. Panel **c** reproduced with permission from AAAS^35^ under a CC BY-NC 4.0 license; Panel **d** adapted with permission from ref. ^23^.

The idea of creating a planar hyperbolic metasurface using a thin metallic grating was explored in ref. ^17^, where it was shown how these structures could be used to control and manipulate surface plasmons in the visible regime. An interesting possibility is to substitute the metal with a 2D semimetal (e.g. graphene) and thus create a hyperbolic metasurface that could operate in other frequency regimes^18^. It would of course be highly desirable if such hyperbolic plasmons can also exist naturally in 2D materials. A number of uniaxial natural 3D hyperbolic materials have been studied to date, including *α*-quartz^19^, hexagonal boron nitride^6,20^, and others^3^. However, the possibility of planar hyperbolicity was speculated to be theoretically possible through the interplay between intraband and interband processes^21^. Since then, a plethora of experiments have uncovered planar hyperbolic polaritons in various 2D materials, with the first report on planar hyperbolic phonon polaritons^9,22^ in *α*-MoO_3_ as illustrated in Fig. 2a–c, followed by similar observations of planar hyperbolic plasmon polariton in WTe_2_^23^ (see Fig. 2d).

### Super-singularity and extreme confinement

Nanoscale light–matter interactions can be tailored through the engineering of the photonic density-of-states or mode volume. The most widely studied among these modes is the plasmon polariton^24^, which possesses dispersion characteristics that are intermediate between those of electrons and photons. While electrons enable highly miniaturized electronic components for computing and memories, photons offer functionalities such as long-range communication links based on silicon photonics. An EM mode that possesses both of these characteristics would be an ideal bridge between electronics and photonics^25^. Other well-known approaches include utilizing Mie resonances and photonic crystals, but these approaches are inherently spectrally narrow. A hyperbolic metamaterial, which is known to possess unusual dispersion relations with open iso-frequency surfaces, as shown in Fig. 2b, allows for access to large wavenumbers across broad spectral windows. Hence, their photonic density-of-states is often dubbed a super-singularity^26^, making possible applications where high optical resolution and sensitivity are desired^27^. The super-singularity is only curtailed by absorption losses in the material and spatial dispersion i.e. non-local response^28^.

However, conventional nanostructured hyperbolic metamaterials and metasurfaces have several important limitations^29^; cutoff in the accessible momenta limited by its physical feature sizes, metallic losses, and the inability for active tuning (for metal-based systems) and difficulty in accessing volume modes for the case of 3D hyperbolic media. Thus, the discovery of natural 2D van der Waals (vdW) materials with natural planar hyperbolic properties provides a significant advance in the effort to create a toolbox for the control of EM fields at the nanoscale.

### Biaxial planar hyperbolic media

vdW materials are generally biaxial media where their principal components of the dielectric tensor *ϵ* are dissimilar. For plane waves e^ı(**k**⋅**r**−*ωt*)^ traveling inside an anisotropic medium, the wave equation can be expressed in the form:

1 $${{{{{{{\rm{k}}}}}}}}\times ({{{{{{{\rm{k}}}}}}}}\times {{{{{{{\rm{E}}}}}}}})+{k}_{0}^{2}\epsilon {{{{{{{\rm{E}}}}}}}} \,=\, 0$$

where E is the electric field, *k* and *k*_0_ are the wavevectors in the medium and free space respectively and *ϵ* is the permittivity tensor.

The governing equation for an isofrequency surface, that is, the allowed *k* vectors for a fixed frequency for plane waves propagating inside a medium, is determined by applying the condition for the nontrivial plane wave solution in Eq. (1). For a diagonal permittivity tensor, this equation is given by

2 $$\left| \begin{array}{cccc}&{\epsilon }_{xx}{k}_{0}^{2}-{k}_{y}^{2}-{k}_{z}^{2}&{k}_{x}{k}_{y}&{k}_{z}{k}_{x}\\ &{k}_{x}{k}_{y}&{\epsilon }_{yy}{k}_{0}^{2}-{k}_{z}^{2}-{k}_{x}^{2}&{k}_{y}{k}_{z}\\ &{k}_{z}{k}_{x}&{k}_{y}{k}_{z}&{\epsilon }_{zz}{k}_{0}^{2}-{k}_{x}^{2}-{k}_{y}^{2}\end{array}\right| \,=\, 0$$

We consider the exemplary anisotropic vdW material *α*-MoO_3_ as illustrated in Fig. 2a. Here, due to translational symmetry in the *xy* plane, one can fix the planar wave-vector and look for allowed *k*_*z*_ solutions. Eq. (2) is, in general, a quartic equation in *k*_*z*_ and is expected to yield four solutions. For a given wavevector, the group velocity of propagation is given by the normal to the isofrequency surface at that point. As a reminder, the isofrequency surface for usual elliptical materials (i.e. 0 < *ϵ*_*x*_ ≠ *ϵ*_*y*_ ≠ *ϵ*_*z*_) consists of two closed surfaces, which in general, intersect at four points along the two optic axes. For biaxial hyperbolic media, however, the isofrequency surface becomes highly nontrivial as shown in Fig. 2b. For example, an isofrequency surface with Re[*ϵ*_*x*_] < 0 < Re[*ϵ*_*y*_], Re[*ϵ*_*z*_] consists of a hyperboloid opening along the *k*_*x*_ direction.

In the extreme limit of 2D planar hyperbolic surfaces, the optical response is dictated by the surface conductivity tensor^21^. In this case, the hyperbolicity arises from the difference in the sign of the imaginary part of the diagonal surface conductivity components^21^. In a slab of planar hyperbolic material, the polariton dispersion is given by^30^

3 $$k(\omega )=\frac{\psi }{t}\left[{\tan }^{-1}\left(\frac{{\epsilon }_{1}\psi }{{\epsilon }_{z}}\right)+{\tan }^{-1}\left(\frac{{\epsilon }_{2}\psi }{{\epsilon }_{z}}\right)+\pi n\right]$$

where *n* is an integer, *t* is the slab thickness, *ϵ*_1_ and *ϵ*_2_ are the permittivities of the surrounding media, *k* is the polariton wave-vector and $\psi=\imath \sqrt{{\epsilon }_{z}/({\epsilon }_{x}{\cos }^{2}\alpha+{\epsilon }_{y}{\sin }^{2}\alpha )}$, *α* being the angle between the *x*-axis and the polariton wave-vector. For propagating modes, one can impose the condition *ℜ*{*k*} > *ℑ*{*k*}. This produces the isofrequency surfaces shown in Fig. 2b. The isofrequency surfaces for 2D polaritons are not merely the projection of the 3D isofrequency surfaces. This is because the refractive index of the surrounding media also influences the 2D polariton dispersion.

### Physical origin of plasmonic hyperbolicty

**Fig. 3:**
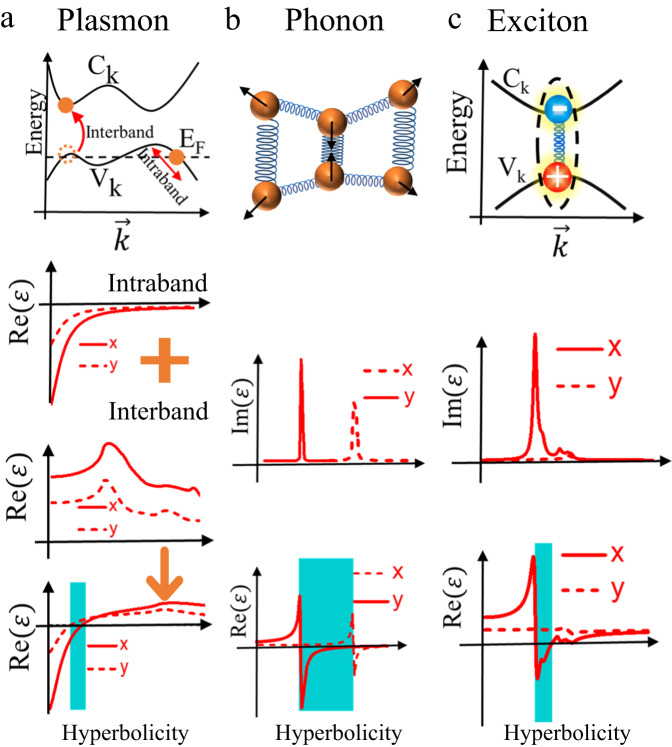
Origin of the planar hyperbolic plasmon, phonon, and exciton polaritonic excitations in archetypal vdW materials, and unifying key features in their dielectric tensors. **a** Planar hyperbolicity generated by plasmon polaritons in the transition metal ditelluride WTe_2_. The hyperbolic region manifested by real parts of permittivities along **x** and **y** directions stems from the interplay of anisotropic intraband and interband transitions^31^, where *V*_*K*_ and *C*_*K*_ represent the valence and conduction bands, and *E*_F_ denote the Fermi energy. **b** Planar hyperbolicity induced by phonon polaritons in a vdW *α*-MoO_3_ flake. The Reststrahlen band originates from the planar phonon mode along crystalline *y* direction^35^. **c** Planar hyperbolicity induced by exciton polaritons in BP. The negative real part of permittivity along the *x* direction results from the strong excitonic resonance due to the strong binding energy of the 2D exciton. The optical dielectric function that involves electron–hole excitations is obtained through solving the Bethe–Salpeter equation. Hyperbolic energy regimes are highlighted with cyan color.

The basic mechanism for hyperbolic plasmon was first theorized as due to the interplay between intraband and interband processes^21^. In metallic systems, the collective motions of free electrons can generate extremely strong optical absorption below the plasma frequency. The *ϵ*_imag_ due to the intraband transition results in a negative *ϵ*_real_ as shown in Fig. 3a. Although anisotropic materials would in general impose anisotropy on *ϵ*_real_, this would not produce hyperbolicity. On the other hand, interband transitions can contribute positive *ϵ*_real_, and its dielectric response is in general anisotropic as dictated by the dipole transition matrix. As a result, hyperbolic dielectric tensors can in principle be induced through the interplay of both intraband and interband transitions, as illustrated in Fig. 3a for the prototypical example of WTe_2_^23,31^.

Since the real (*ϵ*_real_) and imaginary (*ϵ*_imag_) parts of the dielectric function are connected through the Kramers–Kronig relation^32,33^, *ϵ*_imag_ exhibiting a strong peak absorption at *ω*_0_ can result in a negative (or positive) *ϵ*_real_ for frequencies larger (or smaller). Hence, one essential condition to attain a hyperbolic plasmonic surface in a medium with *ϵ*_real,*i*_ × *ϵ*_real,*j*≠*i*_ < 0 is that *ϵ*_imag_ should exhibit strong resonant-like features along at least one of the crystal directions, where subscripts *i* and *j* denote the diagonal elements of the dielectric tensor. The spectral weight of *ϵ*_*imag*_ is characterized by its electric dipole transition matrix elements^31^.

### Planar hyperbolicity via phonons

Optical phonons are quanta of vibrations associated with out-of-phase oscillations of atoms within a unit cell. In polar crystals, such an oscillation can give rise to a net charge dipole that can couple to radiation. It is well-known that the dipole polarizability changes signs as one sweeps the frequency across the dipole resonance frequency^34^. It is this phenomenon that is ultimately responsible for the change in the sign of the dielectric function near the optical phonon resonant frequency, as illustrated in Fig. 3b for the prototypical example of *α*-MoO_3_^35^.

In the context of phonon-based natural planar hyperbolic systems, the crystal anisotropy in the planar direction leads to different optical phonon frequencies in the *x* and *y* directions. The optical dielectric permittivity tensor is then given by a Lorentz model of the form^35^:

4 $${\epsilon }_{k}(\omega )={\epsilon }_{\infty }^{k}\left(1+\frac{{({\omega }_{{{{{{{{\rm{LO}}}}}}}}}^{k})}^{2}-{({\omega }_{{{{{{{{\rm{TO}}}}}}}}}^{k})}^{2}}{{({\omega }_{{{{{{{{\rm{TO}}}}}}}}}^{k})}^{2}-{\omega }^{2}-\imath \omega {\gamma }_{k}}\right)$$

where *k* = *x*, *y* and ${\omega }_{{{{{{{{\rm{LO}}}}}}}}}^{k}$, ${\omega }_{{{{{{{{\rm{TO}}}}}}}}}^{k}$ are the longitudinal and transverse optical phonon frequencies in the respective directions and *γ*_*k*_ is the damping associated with the phonon modes. By definition, a longitudinal (transverse) optical phonon corresponds to the case where the phonon wavevector is parallel (perpendicular) to the direction of oscillation. For frequencies between the LO and TO phonon resonances (called the Reststrahlen band) for say the *x* direction, Eq. (4) shows that the dielectric tensor component *ϵ*_*x*_ becomes negative. If these optical phonon frequencies for the *x* direction are located far away from the ones in the other planar direction *y*, then *ϵ*_*y*_ > 0 and planar hyperbolicity results in this system.

### Hyperbolicity induced by excitons

Unlike plasmon and phonon–polaritons, finite exciton population requires photo-excitation with a pump beam detuned to the red from the optical gap. When the exciton densities are below the Mott threshold for the condensation into electron-hold liquid, the excitons are well described by the Wannier Mott model of Coulomb-bound electron–hole pairs. The dielectric response of the materials will be influenced by the presence of these excitons. Due to the lower dimensionality of vdW materials, dielectric screening is also reduced, allowing a substantial portion of the electric fields within the bound electron–hole pair to permeate outside of the vdW materials, leading to strong exciton binding energies on the order of 100 meV^36^.

The excitons produce resonant absorption peaks in the optical spectra, whose spectral weight is given by the exciton oscillator strength. Since the oscillator strength goes inversely with the effective Bohr radius of the exciton, the strongly bounded excitons in 2D lead to very prominent absorption peaks (or *ϵ*_imag_) compared to their 3D counterparts. Similar hyperbolic dielectric response can might arise in anisotropic 2D semiconducting materials. Figure 3c depicts the calculated optical spectra for monolayer black phosphorus (BP) by means of density function theory in conjunction with solving the Bethe–Salpeter equation for the two-particle Green’s function, which reveals the existence of a hyperbolic window. We shall survey the experiment progress in the observations of these hyperbolic polaritons in the next section.

## Experimental observations of planar hyperbolic polaritons in vdW materials

### Experiments on planar hyperbolic phonon polaritons

Natural planar hyperbolic polaritons were first observed in *α*-MoO_3_ with scattering-type scanning near-field optical microscopy (s-SNOM)^9,22,37^ and photo-induced force microscopy (PiFM)^35^. In these techniques, the polariton is launched by a metallized atomic-force microscope tip, allowing the s-SNOM to detect the scattered light intensity, whereas the PiFM measures the optomechanical force at the tip. The measured interference pattern resulting from the tip-scattered outgoing and field reflected from the flake edge or a highly reflective boundary (e.g. metal structure or defect) reveals the wavevector at the excitation laser frequency by taking a Fourier transform of the lines along the polariton propagation direction^38^, as shown in Fig. 2c. Repeating the process as a function of frequency enables the user to extract the dispersion relationship for the polaritonic modes.

In a follow-up work on *α*-MoO_3_^35^, real space images of the concave wavefront of the planar hyperbolic phonon polaritons, as well as the full dispersion, were experimentally extracted. In this experiment, a silver nanowire was used as an antenna to launch the polariton, thereby replacing the role of the s-SNOM tip in polariton excitation. In practice, both types of excitations still occur, however, typically the scattering of the metal antenna dominates. Furthermore, the polariton lifetime in *α*-MoO_3_ and *α*-V_2_O_5_ were both measured to be on the order of a few ps^35,37^ at room temperature, which is of the same order as high-quality graphene plasmon^39^ and hBN phonon polariton^40^.

### Plasmonic planar hyperbolicity observed

Experimental observation of plasmonic surfaces with planar hyperbolic polaritons was reported in WTe_2_ thin films^23^. As shown in Fig. 2d, the WTe_2_ thin film is in the *T*_d_-type phase. Here, the *a*-axis is defined along the direction parallel to the tungsten chain. The planar anisotropic optical properties of WTe_2_ thin films were originally probed using polarization-resolved far-field optical absorption (or extinction) measurements^23^. A rectangle array along the two optical axes of WTe_2_ films on polycrystalline diamond substrates was fabricated. The ribbon structure provides the required momentum that allows light to couple with the plasmons^41^, revealed as a resonant absorption peak in its extinction spectra. The resonance along the *a* direction is much stronger than that along *b*, and both occur at different resonant frequencies, indicating an anisotropic plasmonic dispersion.

The fitted plasmon dispersion considering only the intraband contributions, follows $\omega \propto \sqrt{q}$, with good corroboration to the measured plasmon peaks at low energy. As the wave vector increases, the dispersions along both planar axes soften and deviate from the $\sqrt{q}$ scaling due to the onset of interband transition. The plasmon polariton is only allowed to propagate along the *a* direction in the frequency range of 429−632 cm^−1^, indicative of a hyperbolic regime governed by the interband transition^21^. The isofrequency contours of the plasmon at different energies reveal the topological transition from the elliptic to hyperbolic regimes, as shown in Fig. 2d. Besides the observed mid-infrared hyperbolicity, monolayer WTe_2_ also exhibits strong anisotropic dielectric tensors at ~1.0 eV, producing a new near-infrared hyperbolicity^31^, which can be ascribed to the band nesting effect.

### Experiments on hyperbolic exciton-polaritons

Due to the contrasting spectral resonance along the crystal axes, planar hyperbolicity is likely to occur around the exciton–polariton frequencies, as suggested by theoretical calculations in Fig. 3c. On the other hand, 3D hyperbolic excitons have been experimentally observed in uniaxial vdW materials. For instance, 2D hybrid perovskites composed of alternating Pb-I octahedral sheets expanding in two dimensions and organic cations have been reported to support hyperbolic exciton polariton in the visible range^42^, and optically induced excitonic hyperbolicity was also discovered in the layered transition metal dichalcogenide WSe_2_^43^. Unlike hyperbolic phonon–polaritons, exciton–polaritons, which originate from electronic processes, can be tuned by varying the optical excitation density. This provides a platform for designing on-demand hyperbolicity at infrared frequencies.

Recent far-field light scattering experiments have extracted the optical constants of monolayer BP, revealing that the dielectric response might be due to hyperbolic planar excitons^8^. From the measured extinction spectra, the dielectric constants along the armchair and zigzag directions can be extracted. In particular, the oscillator strength for the armchair polarization is significantly larger than that in the zigzag, due to the polarization-sensitive nature of the optical transition matrix elements. As a result, a hyperbolic dielectric window was observed across the spectral range from 1.70 to 1.84 eV^8^. This is consistent with first principle calculations shown in Fig. 3c.

The first experimental observation of in-plane hyperbolic exciton-polaritons (HEPs) was reported in the vdW semiconductor chromium sulfide bromide (CrSBr)^44^. In CrSBr, in-plane HEPs exhibit wavefronts that develop with oscillatory out-of-plane electric fields propagating through the bulk-like waveguide modes. The existence of HEPs was confirmed through a combination of energy, temperature, and thickness-dependent measurements. Furthermore, CrSBr is an A-type antiferromagnet material below the Néel temperature *T*_N_ = 132 K, where excitons have been found to couple to magnetic order, providing an additional way to manipulate hyperbolicity by applying magnetic fields.

## Engineering planar hyperbolic materials

### Lower symmetry shear and ghost polaritons

**Fig. 4:**
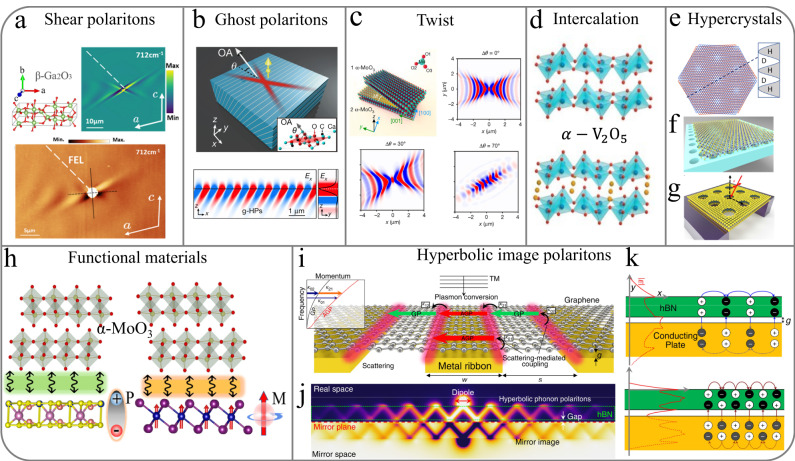
Unique approaches in manipulating hyperbolic polariton properties, which range from exploiting lowering crystal symmetries, crystal orientations, twisting, intercalation, nanopatterning, functional materials, and metal proximity. **a** Control of polariton propagation through hyperbolic shear polaritons in *β*-Ga_2_O_3_. Experimental near-field image and finite element modeling of hyperbolic shear polaritons, revealing the off-diagonal components of the permittivity tensor results in a shear rotation of polariton. The dashed white line is the orientation of the incident free-electron laser (FEL) beam for scanning near-field optical microscopy. **b** Ghost polaritons in uniaxial materials of calcite with the tilted optic axis to the interface, where OA denotes the optic axis, and *θ* is its tilt angle to the surface. Near-field distribution of the ghost polaritons propagating along the surface in the *x*–*z* and *y*–*z* planes. **c** and **d** Phonon polariton modulation via twisted bilayer and atomic or molecular intercalation, where Δ*θ* is the twist angle. **e** Twisted anisotropic 2D vdW layers resulting in Moire pattern. The line cut indicates the regions where one can anticipate hyperbolic and dielectric dispersions. **f** Modulating dielectric substrate to achieve a hypercrystal. **g** Dielectric modulation through direct pattering of the 2D material, where the black dashed lines and *θ* indicate the incidence plane and angle, respectively. **h** Planar hyperbolic phonon polaritons tuned in vdW heterostructure with 2D ferroelectric and ferromagnetic materials. **i** The acoustic plasmon resonator architecture and coupling routes to plasmon modes for a plane wave normally incident with transverse magnetic polarization. **j** Schematic illustrations of image polaritons with launched out-of-phase charge oscillations and radiation confinement to the gap between the hBN layer and the mirror. **k** Charge distributions for symmetric mode and anti-symmetric mode. Panel **a** reproduced with permission from ref. ^11^; Panel **b** reprinted with permission from ref. ^46^; Panel **c** reproduced with permission from ref. ^10^; Panel **d** reprinted with permission from ref. ^37^; Panel **g** reprinted with permission from ref. ^57^; Panel **i** reproduced with permission from ref. ^133^; Panels **j** and **k** reproduced with permission from ref. ^68^.

New opportunities are afforded by pushing to even lower symmetry crystals. While orthorhombic crystals such as *α*-MoO_3_ give rise to highly directional polariton propagation, in monoclinic and triclinic systems, novel hyperbolic shear polaritons can be supported. These modes were first reported using the wide bandgap, monoclinic semiconductor *β*-Ga_2_O_3_^11^ using Otto configuration experiments (Fig. 4a), then imaged in real space in both *β*-Ga_2_O_3_ (Fig. 4a) and another monoclinic material CdWO_4_^45^ via s-SNOM. In triclinic crystals, the three crystallographic axes are not orthogonal, while in monoclinic crystals there is one axis that is not orthogonal to the other two. The implication of this is that the permittivity tensor cannot be diagonalized, and thus, off-diagonal real and imaginary contributions to the tensors are induced. With the monoclinic axis within the plane of the crystal surface (e.g. the (010) surface of *β*-Ga_2_O_3_), the loss of mirror symmetry also results in a breaking of the symmetry in the polariton dispersion (Fig. 4a).

While originally observed within a bulk crystal, exfoliation of *β*-Ga_2_O_3_ is possible, in conjunction with a plethora of monoclinic and triclinic 2D materials. As free-carrier injection into semiconductors can introduce an amplification of off-diagonal loss contribution, it provides a means to dictate the polariton shear and tilted wavefront via electrostatic gating and/or optical pumping to control polariton propagation for on-chip photonics.

The surface of uniaxial crystals whose optic axis is tilted with respect to the surface normal (Fig. 4b) can then accommodate the ghost polariton^46^—the special case of the electromagnetic ghost wave^47^. These ghost polaritons feature a hyperbolic character that is both propagating as well as evanescent in the crystal bulk, leading to slanted phase wavefronts. Engineering the dispersion of ghost polaritons using off-cut calcite crystals has revealed propagation lengths up to 20 μm (Fig. 4b), providing new opportunities for nanoscale wave manipulation^46^.

### Twisted bilayers and magic angles

There has been recent interest in a new type of polaritonic topological transition using twisted bilayers of planar hyperbolic materials^48^. The topological transition from hyperbolic to elliptical states occurs at so-called ‘magic angles’, in close analogy to the newly emerging field of twistronics in condensed matter^49^. The basic principle relies on the variation of the number of crossing points between the hyperbolic isofrequency surfaces of the top and bottom layers as a function of the twist angle^48^. Due to the interaction between polaritons, these degeneracy points in *k*-space create an anti-crossing. The number of these anti-crossing points (*N*_ACP_) governs whether the topology of the isofrequency surface of the twisted bilayer polariton is elliptical or hyperbolic. At the twist angle corresponding to the topological transition (referred to as the ‘magic angle’), the polariton propagation occurs in the canalization regime where a highly directional diffraction-less propagation is observed, as shown in Fig. 4c, realized in *α*-MoO_3_. This phenomenon is topological in the sense that the opening or closing of the isofrequency surface and the magic angle features are not influenced by disorder or imperfections but only by the topological index *N*_ACP_ in *k*-space.

Twisted polaritonics can also inherit other new optical properties. For example, chiral plasmons in twisted atomic bilayers are featured with a unique phase relationship of ±*π*/2 phase difference between their transverse-electric and transverse-magnetic wave components^50^. Such a unique phase relationship for chiral plasmons can lead to unconventional longitudinal spin of plasmons^50^, besides the usual transverse spin of plasmons^51^. Looking forward, it is anticipated that other emergent phenomena might arise from twisted hyperbolic polaritonics.

### Tuning via metal ion intercalations

Recently efforts have also been geared towards demonstrating the active tunability of planar hyperbolic phonon polaritons in 2D materials, with particular emphasis on polariton switching applications. Since the interlayer distance in *α*-MoO_3_ single crystals is quite large, metal ion intercalation offers a potentially effective tuning knob. This technique was demonstrated for the first time via Sn ion intercalation in *α*-MoO_3_^9^. Intercalation can modify the phonon polaritons in three ways. First, this results in lattice distortion, in particular the expansion of the vertical Mo–O bond, which in turn modifies the out-of-plane vibrational modes. Secondly, the Sn ions might act as scattering centers for phonon polaritons resulting in their increased damping. Thirdly, the Sn intercalation results in the introduction of free electronic carriers in *α*-MoO_3_ via doping, potentially enabling the excitation of plasmon modes, which are inherently lossier, as well as the potential for plasmon–phonon polariton coupling. Similar efforts using sodium ion intercalation have also been shown to result in tunable Reststrahlen bands in *α*-V_2_O_5_ (see Fig. 4d) providing frequency shifts as large as 30 cm^−1^^37^, with no significant deterioration of polariton lifetimes being observed. Subsequently, a reversible tuning of these planar hyperbolic phonon polaritons was also demonstrated via hydrogen intercalation in CVD-grown films^52^.

### Hypercrystals

Photonic hypercrystals (PHCs) represent a new class of artificial photonic media distinct from the more widely used systems: metamaterials and photonic crystals^53^. In fact, the PHCs take on the best of both and offer unprecedented control over light propagation and light-matter interactions. The unit cells are sub-wavelength in dimension, yet their response is markedly distinct from the effective medium response seen in metamaterials. This distinction has been exploited to overcome some of the limitations of metamaterials such as out-coupling issues^54^ and the prediction of Dirac physics and singularities^55^.

Hypercrystals in 2D vdW materials can be realized by introducing a periodic modulation of the hyperbolic medium through twisting and dielectric modulation. A schematic of the Moire pattern and the associated modulation of hyperbolic (H) and dielectric (D) regimes are provided in Fig. 4e. Further work is needed to establish the guiding principles governing the tuning parameters to realize such a modulation. Indeed, local changes in the environment^56^, where refraction of hyperbolic phonon polaritons, were achieved using the phase change material VO_2_. In that work, by working close to the phase transition temperature, local domains of dielectric and metallic VO_2_ coexisted, causing the wavevector of the hyperbolic modes to be locally modulated^56^. Further work illustrated the potential of this approach for realizing reconfigurable metasurfaces, via placing the 2D hyperbolic material on a pre-patterned dielectric substrate as shown schematically in Fig. 4f or by directly patterning features into the medium^57^ as shown in Fig. 4g.

### Heterostructures for hybrid polaritons

The advent of vdW materials has enabled the ability to design and fabricate versatile heterostructures with atomic-level precision stacking. In h-BN-based vdW heterostructures, hyperbolic polaritons can be dynamically tuned when placed onto graphene^58^ and phase change materials^59,60^. There have been several recent efforts in achieving the tunability of planar hyperbolic phonon polaritons in *α*-MoO_3_ by coupling them to graphene plasmons^61–63^. On the other hand, the character of plasmon and exciton–polaritons are amenable to the influence of electric fields, via carrier doping or band bending, allowing for dynamic electric control of their ray-like propagations^21^. Such ray-like propagation is at the heart of applications such as hyperlensing^64^ and controllable nanoscale focusing^65^, to be discussed later.

In addition, other emerging physical properties in vdW systems, including superconductivity^49^, magnetism^66^, and ferroelectricity^67^ may also couple with natural hyperbolic polaritons for augmented functionality of these nano-optical modes. Practical examples include natural hyperbolic materials with 2D ferroelectric and/or ferromagnetic substrates illustrated in Fig. 4h, where the strong hysteresis can be exploited for polariton memory. We will revisit these emerging new functional materials in the next section.

### Hyperbolic image polaritons

When polaritons in a vdW material couple with their image charges across a tiny dielectric gap, a virtual mode called an image polariton^68^ can be formed. One possible scheme utilizes a mirror plane that is separated from a vdW film by a nanometer-thin insulator (see Fig. 4i–4k). In this configuration, a polariton mode coupled with its image charge distribution can enhance the field confinement inside the nanometric gap. Image polaritons in vdW materials have been the focus of intense research as they can provide extreme light confinement beyond what is possible with conventional metal-based plasmons. However, such tight confinement leads to large momentum mismatches with free-space photons and also increased optical losses. Hyperbolic image phonon polaritons in hBN can address both of these challenges and have been shown to exhibit effective refractive index of up to 132 and quality factors as high as 500 in isotopically enriched hBN^68^.

The key advantage of this approach is that unpatterned pristine vdW materials can be used in contrast to other resonator designs based on patterned vdW structures. Since 2D polaritons are tightly confined, it is crucial to minimize unwanted surface roughness and etch-induced damage during the fabrication process. The fabrication of the multi-layer substrate, which includes metal ribbons, an optical spacer, and a reflector, was accomplished via a template-stripping process^68^ to create ultraflat metal surfaces^69^. The optical spacer and the reflector further boost the coupling efficiency by recycling the transmitted waves back to the image polaritons when the quarter-wavelength condition is satisfied. A similar strategy can be used to harness planar hyperbolic image polaritons toward efficiently launching ultra-low-loss and ultra-compressed polariton modes.

## Functional hyperbolic materials

**Fig. 5:**
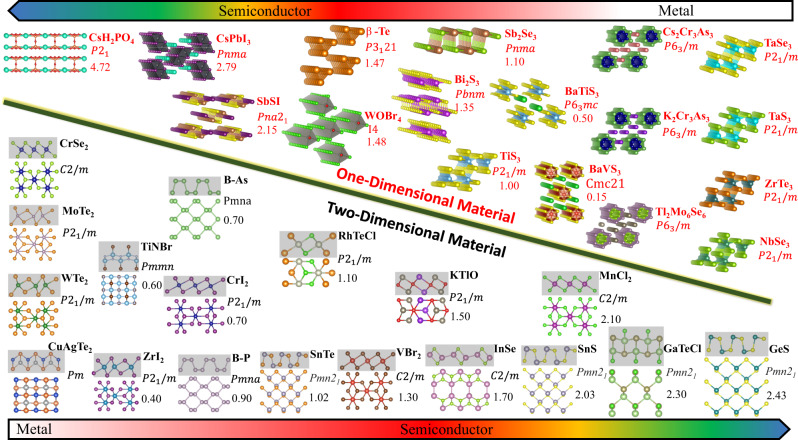
Untapped opportunities of synergizing hyperbolic polaritons in low symmetry biaxial or uniaxial vdW materials with functional properties ranging from ferromagnetism, ferroelectricity and piezoelectricity. Selected 1D and 2D materials with structural anisotropy potential to display the hyperbolicity. Both 1D and 2D materials are chosen from experimentally known compounds; only planar anisotropic 2D materials are considered in this plot. Chemical formula, space group and bandgap (in eV) are listed for each material. Arrangement is based on their bandgap, followed by the top and bottom color bars measuring the bandgap scale. For each 2D material plot, the top gray shaded region and bottom part display the crystal structure from side and top views, respectively.

Besides the foregoing 2D materials, we anticipate many potential candidates that exhibit planar hyperbolicity in the class of vdW materials. Selected 2D materials with planar structural anisotropy are shown in Fig. 5, which have all been exfoliated from their known bulk crystal form^70^. More interesting are the diverse functional properties that many of these materials possess, such as ferromagnetism, ferroelectricity, piezoelectricity and thermoelectricity. In Fig. 5, we sort these materials according to their bandgaps that span across the metallic to semiconducting and insulating range, potentially hosting various polaritons across different spectral ranges.

### Ferromagnetic biaxial vdW crystals

Ferromagnetic orderings offer a novel route to modulating the plasmonic modes through magneto-optical effects^71^ and open up the possibility of non-volatile tuning of plasmon polaritons via the magnetization induced in the host material. As experimentally observed, the plasmon-polariton wavevector can be effectively modulated by switching the direction of the magnetization^71^. Most notably, magnetized materials have been demonstrated to enable breaking of time-reversal symmetry to introduce non-reciprocity^72^. Moreover, excitation of the surface plasmon polariton was also shown to depend on the magneto-optical properties of the medium^73^. Magneto-optic and non-reciprocal effects with magnetic materials offer broad promise for enriching the physics of hyperbolic polaritons such as enabling the selective directional control of the hyperbolic rays^74^.

As shown in Fig. 5, CrSe_2_, VSe_2_, CrTe_2_, VBr_2_, and FeCl_2_^70^ are 2D ferromagnetic materials and adopt the structure characterized by edge-sharing octahedra^75^. Their frequency-dependent dielectric functions have been found to display linear optical birefringence^76^. CrI_2_ is also a vdW ferromagnetic material with the same 1T${}^{{\prime} }$ crystal structure as WTe_2_. It is antiferromagnetic at the monolayer limit, but it can transition to ferromagnetic as the layer thickness increases due to interlayer vdW interactions^77^. It is worth mentioning that most of the 2D ferromagnetic materials possess relatively low intrinsic Curie temperature, but monolayer 1T-VSe_2_ has been found to retain ferromagnetic ordering above room temperature^78^. These new magneto-optic materials, with their potential for hyperbolic polaritons, can be the basis for a new class of polaritonic memories.

### Ferroelectric biaxial vdW crystals

Ferroelectric materials are known to host soft optical phonon modes accompanied by large dipole moments. Hence, anisotropic vdW ferroelectric materials offer fertile ground for tunable hyperbolic polaritons in the far infrared^79^. GeS, SnS, GeSe, SnSe, and GaTeCl^80^ have all been predicted to display spontaneous room-temperature ferroelectricity in the monolayer limit based on first-principle simulations. Subsequently, ferroelectricity at the monolayer limit in SnTe, SnS, and SnSe were confirmed through electron microscopy images and spatially resolved differential conductance spectra^81,82^. In particular, the robust planar polar distortion^80,83^ in these materials is responsible for the structural, electronic, and optical planar anisotropy^84^.

Phonon polaritons in ferroelectric materials should in general exhibit wider Reststrahlen bands, owing to the larger splitting between the longitudinal and transverse optic phonon modes^79^. This is the result of the large Born effective charge associated with the enhanced polarization in ferroelectric materials. A larger Reststrahlen band would imply hyperbolic phonon polaritons that can be supported over a larger spectral bandwidth. In a similar fashion to magnetic materials, the polariton dispersion and damping are observed to be strongly influenced by the polar phonons, due to the cross-anharmonic couplings between ferroelectric modes and normal-mode lattice vibrations^79^. In addition, the remnant ferroelectric polarization can also drive carrier modulation in a semimetal 2D layer on a ferroelectric substrate via electrostatic doping^85^. Hence, both plasmon and phonon polaritons are controllable with applied electric fields, mediated by the polarization modulation.

### One-dimensional vdW materials

We also searched the class of quasi-one-dimensional (quasi-1D) materials, as illustrated in Fig. 5. In these materials, the atoms are strongly bonded by covalent or ionic bonds along the intrachain direction but mediated by weak vdW forces along the interchain direction, which results in significantly anisotropic electronic, and structural properties. For example, K_2_Cr_3_As_3_^86^, Cs_2_Cr_3_As_3_^87^ and Tl_2_Mo_6_Se_6_^88^ were experimentally found to possess quasi-1D ferromagnetic ordering and display anisotropic superconductivity, magnetoresistance, and electronic transport. A series of quasi-1D ferroelectric materials BaTiS_3_^89^, BaVS_3_^90^ and WOBr_4_^91^ have been found to develop robust ferroelectric distortions within each chain unit.

Similar to the aforementioned functional 2D vdW materials, the quasi-1D ferromagnetic, ferroelectric and piezoelectric materials may exhibit even stronger anisotropic optical properties^89^. Moreover, another possible hyperbolic quasi-1D material is the two-dimensional ordered carbon nanotube film that has been crystallized through a simple vacuum filtration technique^92^. Low-dimensional plasmons originated from longitudinal charge oscillations enable high-quality terahertz and infrared optical applications at deep subwavelength scales^93^. Metallic single-walled carbon nanotubes were also experimentally observed to develop Luttinger-liquid plasmons that exhibit extraordinary spatial confinement and high-quality factor^94^.

## Potential applications

### Quantum and spin photonic applications

For applications such as quantum photonic nodes, highly efficient coherent energy transfer and quantum simulators^95,96^, engineering the interaction between multiple quantum emitters is a key prerequisite^95^. Here, 2D planar hyperbolic polaritons are uniquely suited as reconfigurable one-dimensional waveguides mediating long-range dipole–dipole interactions and entanglement^97^. This has interesting implications for developing on-chip architectures for quantum information processing, significantly reducing the fabrication constraints.

**Fig. 6:**
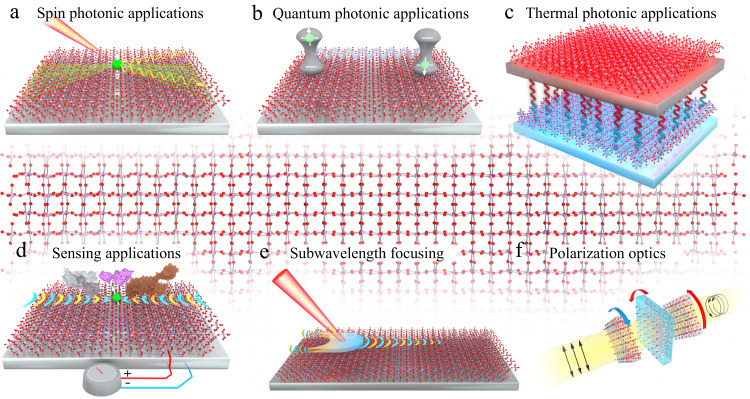
Repertoire of potential applications enabled by planar hyperbolic polaritons made possible by their highly energy efficient energy transfer, ray-like propagation, extreme birefringence, sensitivity to the environment, subwavelength light confinement, and large photonic density-of-states. **a** Spin-momentum locking of evanescent electromagnetic waves in 2D hyperbolic materials. **b** Interaction of two quantum emitters via super-Coulombic dipole–dipole interactions within the 2D planar hyperbolic medium. **c** Near-field radiative heat transfer between a pair of hyperbolic sheets. **d** Biosensing with tunable, plasmonic vdW materials. **e** Infrared near-field optical nanoimaging of *α*-MoO_3_ using a plasmonic antenna. **f** Manipulation of light polarization states using twisted stacks of anisotropic vdW materials.

Recent studies have shown that the light emission of a quantum emitter placed atop a 2D hyperbolic (Fig. 6a), can be selectively channeled into particular hyperbolic modes through prudent control of the emitter polarization^74^. For efficient routing of the quantum emitter spin states into these photonic modes, an important direction to pursue is in the development of 2D hyperbolic material-based devices that can exhibit a helicity-dependent response^98^.

In free space, the spin of the photon is always collinear with the direction of propagation. For surface waves on isotropic 2D materials, due to the electric (TM) or magnetic (TE) fields rotating in the plane perpendicular to the interface, the spin angular momentum is instead transverse to the wave vector^99^. When two quantum emitters are placed near a 2D material, they interact via surface waves supported by this material. However, due to the spin-momentum locking of these surface waves, such interaction will depend on the orientation of the spin of these quantum emitters and will therefore be asymmetric due to the coupling with the momentum direction. A critical and unique functionality is offered by 2D hyperbolic materials—the ability to electrically tune the spin angular momentum of surface waves from transverse to longitudinal^99^. Figure 6b depicts the vision of the dynamical control of the interaction in say, one-dimensional arrays of quantum emitters^100^ placed on the surface of these 2D hyperbolic materials—a key platform for the realization of quantum information processing protocols^100,101^.

### Thermal photonic applications

With the miniaturization of electronic circuits, the heat transfer between two components in the sub-wavelength vicinity of each other will strongly deviate from Stefan–Boltzmann law^102^. The control of radiative heat flow at the nanoscale is an important area of application for 2D materials^103^. Near-field radiative heat transfer via surface EM modes enables extremely efficient energy transport exceeding the black body limit^104,105^. The driving applications include high-temperature coatings, near-field thermophotovoltaics, radiative cooling and heat sinks^106^. The use of hyperbolic phonon polaritons might lead to super-Planckian near-field thermal emission^103^, a subject that is of great current interest^107^. In recent work, systematic thermal transport measurements confirm the remarkable heat conduction mediated by surface phonon polaritons in SiC nanowires^108^. One tool for observing and measuring this effect would be near-field thermal emission spectroscopy where a near-field heated tip thermally excites hyperbolic modes and studies their spectral distribution (Fig. 6c).

Comparing the near-field radiative heat transfer between two uniaxial hyperbolic crystals and two biaxial hyperbolic materials has revealed the superiority of the latter case. For instance, it has been shown that the near-field radiative heat flux between two *α*-MoO_3_ flakes at a vacuum gap of 20 nm is about 2200 kW m^−2^, which is an order of magnitude larger than the flux between two hBN crystals by an order of magnitude^109^. Twist engineering of planar hyperbolic materials would also offer interesting avenues to near-field thermal effects. In the far field, super-Planckian thermal emission is not possible, however, 2D planar hyperbolic media could provide unique spin-resolved thermal emission^110^, an effect that has yet to be observed experimentally.

### Sensing applications

The vibrational spectrum of a substance in the mid-infrared range provides a unique fingerprint enabling its chemical identification. As a rule of thumb, the stronger the polaritonic field confinement, the more sensitive the sensor is to minute changes in the nearby dielectric environment. 2D materials provide a very promising platform for sensing as they support extremely confined polaritons^111^. Moreover, many 2D polaritons appear in the mid-infrared regime (3–20 μm), where most molecular vibrational or rovibrational (for gases) excitations occur (Fig. 6d). Since in planar hyperbolic materials, the polaritons can be guided along effective 1D channels, these could be used to sense molecules at specific locations on the 2D sheet. In principle, it is possible to have an actively tunable sensor that can provide location-specific information about the target molecule orientation and position, as well as their identification^112,113^. Since the quasi-transverse magnetic hyperbolic mode is much more strongly confined compared to quasi-transverse electric modes^114^, the light–matter interaction is dominated by the former.

Recently, materials that support hyperbolic phonon polaritons (like h-BN) are being considered, due to their higher quality factor and the potential of ultra-high EM confinement. In a recent work^115^, arrays of hBN resonators (made with monoisotopic boron (B_10_ or B_11_) to reduce isotope scattering), were covered with a 10 nm thin layer of organic molecules 4,4${}^{{\prime} }$-bis(N-carbazolyl)-1,1${}^{{\prime} }$-biphenyl(CBP), that features a C–N vibrational absorption band at 1450 cm^−1^, which is within one of the h-BN Reststrahlen bands. Moreover, the experiments showed that CBP vibrations and h-BN polaritons were in a strong coupling regime. On the other hand, twisted bilayers of 2D hyperbolic materials can also be an appealing platform for near-field chiral plasmons and chiral sensing, since the physical chirality naturally breaks all mirror plane symmetry. Theoretical studies in twisted bilayer graphene suggest strong near-field chirality^116^.

### Subwavelength focusing and transformation optics

A unique type of subwavelength focusing (Fig. 6e) via planar hyperbolic materials was proposed^35^. Simulations have shown^35^ that depending on the polarization of the excitation beam, the image is composed of anisotropic wavefronts, whose shape is determined by the group velocity direction of the isofrequency surface. While such phenomena can be observed with elliptical materials as well, the subwavelength resolution offers a unique capability of hyperbolic systems as shown in Fig. 6e. Such polarization-dependent focusing could lead to new applications in light trapping and photodetection^117,118^. Recently, there have also been demonstrations of subwavelength focusing of planar hyperbolic polaritons, where using *α*-MoO_3_, as a polaritonic hyperlens, a resolution of *λ*_0_/50 and a bend-free refraction was shown^119^, where *λ*_0_ is the free space wavelength.

Graphene was the first 2D material that was shown to be an excellent candidate for realizing transformation optics devices owing to the gate tunability of its optical conductivity with nanoscale precision^120^. The field has its origins in Einstein’s general theory of relativity, where the form invariance of Maxwell’s equations under coordinate transformations can be represented as changes in the permittivity and permeability tensors^121^. Hence it is intriguing to be able to realize tunable and anisotropic forms of these constitutive parameters for universal transformation optics.

### Polarization and emission engineering

Planar hyperbolic media are most relevant in the development of ultrathin polarization controllers as shown in Fig. 6f^122–124^. Size is a bottleneck in traditional polarization controllers, for instance, a state-of-the-art quarter-wave plate uses bulky linear birefringent crystals, since it requires a significant propagation distance to establish the phase difference between orthogonal polarizations. Recent work has shown that stacks of anisotropic vdW materials can facilitate the building of optical elements with Jones matrices of unitary, Hermitian, non-normal, singular, degenerate, and defective classes. These twisted stacks with electrostatic control can function as arbitrary-birefringent wave-plates or arbitrary polarizers with tunable degrees of non-normality, which in turn gives access to a plethora of polarization transformers including rotators, pseudorotators, symmetric and ambidextrous polarizers^125^.

An interesting application of planar hyperbolic materials is in the development of nonreciprocal spontaneous emission enhancement, opening the door for non-inverse dynamics between degenerate transitions in quantum emitters and coherence generation via anisotropic vacuum^126^. In the context of valleytronics^127^, it is known that the two valleys in gapped Dirac materials emit circularly polarized photons with opposite helicity. However, if these two valleys are not coherently excited, the degree of linear polarization at the output can be very low^128^. In order to overcome this problem, it has been shown that placing these gapped Dirac materials in the near field of planar hyperbolic materials such as *α*-MoO_3_ can result in the spontaneous generation of coherence between the two valley states without the requirement of any external coherent laser source for excitation^62,129^. This is an outcome of the extreme anisotropy of planar hyperbolic materials, which makes the EM vacuum anisotropic resulting in modifications of the respective spontaneous emission rates^130^.

## Conclusions and outlook

In summary, planar hyperbolic polaritons in vdW materials represent an interesting new frontier in nanophotonics, featuring highly directional and confined EM modes with ray-like character on a planar medium allowing for active tunability and ease of coupling to these modes from free space. Planar nanophotonics is appealing as today’s silicon electronics and photonics are based on planar technologies. Looking forward, we have identified the vast engineering space of these planar hyperbolic modes, their new and emerging optical physics, their most promising and interesting applications, and the untapped opportunities that new functional vdW materials can offer to this field. In fact, planar hyperbolic polaritons should be the norm in low-symmetry biaxial or uniaxial vdW materials, and polaritons in many of these materials have yet to be explored. Since these polaritons generally reside in the infrared, access to large areas of vdW materials is essential for most polariton experiments. Polaritonic loss remains an outstanding issue and should be addressed for practical applications. Besides continual improvements in vdW materials growth quality, loss mitigation strategies such as isotropic engineering^131^ and gain media^132^ are viable approaches.
